# Multicentre oncology clinical trials in primary health care in Cuba: evaluation of programme implementation in Villa Clara Province, 2010–2020

**DOI:** 10.3399/BJGPO.2021.0165

**Published:** 2022-07-27

**Authors:** Geidy Lorenzo, Ramón Alberto Ortiz, Rayza Méndez, Migdalia Rodríguez, Rayza Marrero, Nieves del Carmen de la Barca, María Cecilia Granela, Maritza Artiles, Menelio Norvel, Meylan Cepeda, Elsa Toledo, Alexis González, Juan Carlos Crespo, Olga Torres, Leisy Nieto, Tania Crombet, Liset Sánchez, Agustín Lage

**Affiliations:** 1 Center of Molecular Immunology, Havana, Cuba; 2 “Celestino Hernández Robau” Hospital, Villa Clara, Cuba; 3 National Coordinating Centre of Clinical Trials, Villa Clara, Cuba; 4 Chiqui Gómez Polyclinic, Vila Clara, Cuba; 5 Roberto Fleites Polyclinic, Villa Clara, Cuba; 6 XX Aniversario Polyclinic, Villa Clara, Cuba; 7 Marta Abreu Polyclinic, Villa Clara, Cuba; 8 Center of Molecular Immunology, Havana, Cuba; 9 Celestino Hernández Hospital, Villa Clara, Cuba; 10 Central University of Las Villas, Villa Clara, Cuba

**Keywords:** clinical trials, primary health care, oncology, immunotherapy, neoplasms, general practitioners, primary healthcare

## Abstract

**Background:**

The Center of Molecular Immunology of Cuba has developed a programme for the conducting of multicentre oncology clinical trials in primary healthcare centres since 2009.

**Aim:**

To evaluate the ability to conduct oncology clinical trials in primary health care.

**Design & setting:**

A longitudinal, prospective, analytical study was developed between July 2010 and August 2020 in the Villa Clara province.

**Method:**

Structure, process, and outcome indicators were evaluated by the methods of a structured interview, direct observation, documentary observation, and databases analysis. The investigators' curricula vitae, the investigator site file, minutes of workshops, the monitoring reports, the clinical trial training records, and databases were employed as sources of information. The following criteria were considered adequate: when the indicator met the standard; and not adequate: when the indicator did not meet the standard.

**Results:**

The six structure indicators reached adequate results and showed that the programme has allowed building of capacities to conduct clinical trials in primary care. The eight processes indicators and two outcome indicators were considered adequate too. Trials conducted in primary care showed better indicators of patient recruitment than secondary care. Both scenarios showed similar behaviour for the process indicators: retention, protocol compliance, and safety. Survival and satisfaction with health services were also comparable in both scenarios.

**Conclusion:**

The evaluation of the programme showed adequate indicators for conducting oncology clinical trials in primary care in Villa Clara and these were comparable to those determined in the secondary care.

## How this fits in

Oncology clinical trials are mainly developed in specialised medical care centres. The Center of Molecular Immunology in Cuba has developed immunotherapies for the treatment of cancer, with proven safety and efficacy in previous phase I, II, and III studies, developed in hospitals. The safety profile of these products allows their use in primary care institutions, so scientific evaluation is necessary for this context through clinical trials, to allow their subsequent introduction as part of routine medical practice.

## Introduction

The life expectancy of patients with cancer used to be very limited, but in recent decades this picture has changed and, for some types of cancer, prolonged survival rates are observed, reflecting the combined effects of earlier diagnosis, new treatments, and better care.^1^ Advances in personalised medicine and immuno-oncology have demonstrated therapeutic efficacy in several types of cancer and, at the same time, lower toxicity than classical cytostatics, characteristics that allow the use of these therapies for long periods, even beyond disease progression.^2,3^ These two elements have indicated the need to pay greater attention to the follow-up of patients with cancer in primary health care (PHC), which is an ideal setting for the care of patients with chronic conditions.^4,5^ In addition, research conducted in this setting is becoming more relevant, allowing studies to recruit a real-world patient population that is not well represented in clinical studies conducted under controlled hospital conditions.^5^

The Center of Molecular Immunology in Havana, Cuba, has developed several innovative immunotherapies for the treatment of cancer. These innovative molecules have demonstrated their efficacy and safety in phase I, II, and III clinical trials, developed in hospitals across the whole country and abroad. The optimal use of these products implies repeated doses and prolonged administration. These elements raise the possibility of administering these therapies in the PHC setting.^6,7^

The first experience in the country began with clinical trials in PHC with the CIMAvax-EGF vaccine, registered for the treatment of adult patients with advanced lung cancer.^8,9^ Its treatment scheme comprises four induction doses administered every 14 days and subsequently, maintenance doses every 28 days for as long as it is tolerate by the patient.^10,11^

In Cuba, there was no previous experience in conducting clinical trials with products for cancer treatment in PHC centres, so it was necessary to design and implement a clinical trial programme adapted to the conditions of this context.

The aims of the present work were to evaluate the ability to conduct oncology clinical trials in PHC, based on the experience of the CIMAvax-EGF vaccine in Villa Clara province.

## Method

### Population

The central Cuban province of Villa Clara is composed of 13 municipalities and in 2019 had a population of 777 500 inhabitants, with a population density of 92.4 inhabitants per km^2^.^12^ Malignant tumours were the second leading cause of death in 2019, with 1696 deaths and a rate of 218.2 per 100 000 inhabitants. Specifically, trachea, bronchus, and lung tumours ranked first in mortality, with 5626 deaths in 2019.^13^

The oncology services are provided at the Celestino Hernández Robau Hospital, which provides regional services to the provinces of Villa Clara, Cienfuegos, and Sancti Spíritus and covers an estimated population of 2.9 million inhabitants.

The province has 37 primary care units, called ‘polyclinics’, in 18 municipalities. The clinics belong to the primary care system that concentrate services of the main medical specialties and are located in each Cuban community. The clinics' main objective is to provide a comprehensive health service to all inhabitants. Villa Clara has 7499 physicians, which represents 9.65 per 1000 inhabitants; 1753 of these are GPs located in the community.^13^

### Study design and data sources

A longitudinal, prospective, analytical study was developed between July 2010 and August 2020, during conducting two open-label, uncontrolled, non-randomised, multicentre, phase IV clinical trials. These trials were carried out to evaluate the CIMAvax-EGF vaccine in PHC institutions and were registered in the National Public Registry of Clinical Trials (https://rpcec.sld.cu/) — with trial numbers RPCEC00000181 (secondary identifying number IIC RD-EC-120) and RPCEC00000205 (secondary identifying number IIC RD-EC-157), respectively. Trial number IIC RD-EC-157 was ongoing, so, the database cut-off was performed on 31 August 2020.

The first trial was a pilot experience in six polyclinics in the municipality of Santa Clara, the capital area of the province. The second trial expanded the programme to the entire province and covered 17 polyclinics. This trial incorporated the evaluation of EGF concentration as a predictive biomarker of therapeutic success with the CIMAvax-EGF vaccine.

Qualitative and quantitative methods were used to evaluate the programme. The structure, process, and outcome indicators, as well as the instruments used in the evaluation for the collection of information and their comparative standards, were previously validated through expert consensus using the Delphi modification methodology.^14,15^ An exhaustive content analysis of all documents produced by the different institutions and actors involved in the clinical trials was analysed. These documents comprised the investigators' curriculum vitae, the investigator site file (ISF), minutes of workshops, the trial monitoring reports, and the provincial clinical trial training records.

Structural indicators included infrastructure, material, and human resources used in the conduct of clinical trials. Process indicators examined whether the clinical trial programme was conducted as planned. The outcome indicators were designed to assess the effects of the programme on the patients' health status (survival) and the degree of patient satisfaction with the care received (Supplementary Table S1).

For 'survival', the databases of the clinical trials were used. Patients with non-small cell lung cancer, verified by histology or cytology, stage IIIB or IV, performance status 0–2, stable disease, or response to first-line oncology treatment, were included in the analysis.

The indicator 'satisfaction with health care' was evaluated in this subset of patients. The instrument used was the IN-PATSAT32 questionnaire from the European Organisation for Research and Treatment of Cancer (EORTC).^16^ The questionnaires were given to the patients 3 months after the beginning of the treatment with the immunotherapy by the psychologist of the investigational team, with prior informed consent of the patient. Question number 21 was omitted because it was not applicable in this context. For this indicator, a cross-sectional comparison was made with patients included in clinical trials with CIMAvax-EGF in the hospital, in the same period.

For the evaluation of the programme implementation, the indicators were compared with the comparative standards validated by experts. The criteria for the indicators being considered adequate was when they met the standard, and not adequate was when the indicator did not meet the standard (Supplementary Table S1).

Additionally, the results were compared with the indicators obtained in the previous CIMAvax-EGF open-label, controlled, randomised, phase III trial conducted in the hospital (RPCEC00000161, secondary identifying number IIC RD-EC081) considered as historical control (external and non-concurrent). The general scheme of the study was shown in Supplementary Figure S1.

### Statistical analysis

All Villa Clara patients included in the databases of each trial were considered. SPSS (version 19) was used for statistical analysis. The process and outcome indicators were calculated considering the formula defined for each one (Supplementary Table S1). To evaluate overall survival, the primary variable was the survival time measured from the date of the inclusion in the trial to the date of death of the patients or the date of the latest news. Survival times of all patients were estimated using the non-parametric Kaplan–Meier estimator. Median survival and its confidence intervals were estimated. Survival curves between trials were compared using log-rank two-tailed test, considering a significance level of *P* = 0.05.

For 'satisfaction with care' analysis, the mean and standard deviation were calculated and comparisons using *t*-test for independent samples were made. A level of statistical significance of *P* = 0.05 was considered.

## Results

### Structure indicators

For the five structure indicators, the evaluation of 'not adequate' was obtained during the conduct of the first clinical trial protocol (IIC RD-EC-120), developed in the period 2010–2015. These were worse than the proposed standard for the creation of capacities for conducting clinical trials in 90% of the municipalities of the province. However, the indicator 'percentage of professionals and technicians trained in clinical trials and oncology' obtained the qualification 'adequate' (Table 1).

**Table 1.** Indicators of structure in the conduction of multicentre oncology clinical trials in primary care institutions. Villa Clara province, 2010–2020

Indicators	Structure indicators			
	2010–2015 periodClinical trial IIC RD-EC120	Evaluation	2016–2020 periodClinical trial IIC RD-EC-157	Evaluation
1. Percentage of polyclinics conditioned to conduct clinical trials in the province	**33% (6/18**)(6 clinical trial consultations, 6 locals for product administration, 6 pharmacies, 6 laboratories)Certification of compliance of good clinical practices (GCP)	**Not adequate**(<90%)	**94% (17/18**)(17 clinical trial consultations, 17 locals for product administration, 17 pharmacies, 17 laboratories)Certification of compliance of GCP	**Adequate**(>90%)
2. Percentage of municipalities participating in the clinical trials programme	**7.6% (1/13**)	**Not adequate**(<90%)	**92% (12/13**)	**Adequate**(>90%)
3. Percentage of completed investigation teams	**33%** (**6/18**) clinical research teams composed of: 6 primary care physicians, 6 nurses, 6 pharmacists, 6 specialists in laboratory, 6 psychologists	**Not adequate**(<90%)	**94%** (**17/18**) clinical research teams composed of: 17 primary care physicians, 17 nurses, 17 pharmacist, 17 specialists in laboratory, 17 psychologists	**Adequate**(>90%)
4. Percentage of professionals and technicians trained in clinical trials and oncology	**100%** (**337/337**)Courses on GCPs, adverse events, pharmacy, clinical laboratory, nursing83 primary care practitioners trained in oncology (37 general doctors, 37 nurses, and 9 other professionals)	**Adequate**(100%)	**100%** (**373/373**)Courses (GCP, adverse events, pharmacy, clinical laboratory, nursing)	**Adequate**(100%)
5. Percentage of polyclinics with electronic data management	**0%**(Paper data collection)	**Not adequate**(<100%)	**100%** (**17/17**)(Training and certification of 68 users)	**Adequate**(100%)
6. Percentage of polyclinics with standard operating procedures (SOPs) implemented	**0%**(SOPs designed for hospitals was used)	**Not adequate**(<100%)	**100%** (**17/17**)(SOPs designed for primary health care centres)	**Adequate**(100%)

For the second protocol (IIC RD-EC-157), the six indicators showed adequate results, reaching 90% of the municipalities in the province incorporated into the programme and 100% of the human resources trained. In addition, the implementation of electronic data management and standard operating procedures adapted to PHC institutions is highlighted (Table 1). The results showed the expansion of the programme and the continuous improvement of the implementation process.

### Process indicators

The centralised ethics committees allowed obtaining approval time for the protocol and its modifications in <30 days, similar to those achieved in secondary care (Table 2).

**Table 2.** Process indicators in multicentre oncology clinical trials conducted in primary health care in Villa Clara province. Comparison of primary health care versus secondary health care

	Secondary heath care	Primary health care (clinical trial programme implementation)		
	2006–2011 period(phase III, EC IIC-RD-EC081)*n* = 29	2010–2015 period(phase IV, EC IIC RD-EC120)*n* = 183	2016–2020 period(phase IV, EC IIC RD-EC-157)*n* = 127	2010–2020 period*n* = 310
**Study details**	Histological confirmation of NSCLC stage III or IV, **Objective Clinical Response after oncospecific treatment, performance status ≤2**, life expectancy ≥3 months	Histological confirmation of NSCLC stage III or IV, patients classified as non-tributary of any oncospecific treatment or who have received first line of chemotherapy for the advanced disease and do not have not any therapeutic alternatives, **performance status ≤3**, life expectancy ≥3 months	Histological confirmation of NSCLC stage III or IV, patients classified as non-tributary of any oncospecific treatment or who have received first line of chemotherapy for the advanced disease and do not have not any therapeutic alternatives, **performance status ≤3**, life expectancy ≥3 months. **Biomarker determination**	
**Ethical approval**				
7. Time for protocol and protocol modifications ethical approval	Protocol ethical approval:18 daysModification 03: 7 daysModification 04: 16 days	Protocol ethical approval: 28 days^a^Modification 01: 29 days^a^	Protocol Ethical aproval: 22 days^a^Modification 01: 4 days^a^Modification 04: 12 days^a^	
**Recruitment and retention**				
8. Province recruitment versus total trial recruitment	5.8%(29/497)	17.3%^a^(183/1058)	17.1%^a^(127/741)	17.2%^a^(310/1799)
9. Proportion of patients recruited or incidence of disease	2.3%29/1214	27.8%^a^183/658	8.6%^b^127/1463	14.6%^a^310/2121
10. Inclusion rate (patients included per month)	0.36(29/79)	6.5^a^(183/28)	2.6^b^(127/48)	4.0^a^(310/76)
11. Percentage of randomised participants who have withdrawn consent to continue in the study	10.3%(3/29)	9.2%^a^(17/183)	10.2%^a^13/127)	9.6%^a^30/310
**Protocol compliance (adherence**)				
12. Percentage of protocol deviations (non-compliance with treatment scheme)	6.8%(2/29)	4.3%^a^(8/183)	7.0%^a^(9/127)	5.4%^a^(17/310)
**Management of adverse events**				
13. Number of grade 3–4 related adverse events per number of enrolled participants	6.8%(2/29)	2.1%^a^(4/183)	1.5%^a^(2/127)	1.9%^a^6/310
14. Number of serious related adverse events reported per number of enrolled participants	6.8%(2/29)	3.8%^a^(7/183)	0.7%^a^(1/127)	2.5%^a^8/310

Indicator evaluation: ^a^adequate, ^b^not adequate. NSCLC = non-small cell lung cancer

Other study details are available at: http://rpcec.sld.cu/trials/RPCEC00000161-En, http://rpcec.sld.cu/trials/RPCEC00000181-En and http://rpcec.sld.cu/trials/RPCEC00000205-En.

For the 'province recruitment versus total trial recruitment' indicator, a proportion of 17.2% was obtained for the studies conducted in PHC versus 5.8% for the trial conducted in the hospital. Likewise, the 'proportion of recruited patients in relation to the incidence of the disease' was higher in the trials conducted in PHC with 14.6% compared with secondary health care (SHC), where only 2.3% was found. The same behaviour was obtained for the indicator 'inclusion rate' where an increase from 0.36% in SHC to 4.0% in PHC was observed (Table 2).

The 'percentage of randomised participants who have withdrawn consent to continue in the study' indicator showed similar results in both scenarios, with values of 10.3% and 9.6% for SHC and PHC, respectively. In the same way, adherence was similar too, with percentages of 6.8% and 5.4% of non-compliance with the treatment scheme for secondary and primary care, respectively (Table 2).

For the indicator 'number of grade 3–4 related adverse events per number of randomised participants', in PHC units 1.9% was determined versus 6.0% in SHC. Likewise, the 'percentage of related serious adverse events' was lower for the trials conducted in PHC (2.5%) than in the trial conducted in SHC, which was 6.0%. All identified adverse events were consistent with those found in previous trials (Table 2).

The eight process indicators in PHC met the comparative standard, for which they were evaluated as adequate (Table 2).

### Outcome indicators

Median survival of 10.46 (8.44–12.48) and 9.86 (7.11–12.62) months respectively, were obtained for the trials conducted in PHC (IIC RD-EC120 and ICC RD-EC-157), which were similar (*P* = 0.907) to the median of 10.40 (4.85–15.94) found in the previous phase III clinical trial conducted in the hospital (IIC RD-EC081). The 12-month survival rates were 43.1 and 44.0 for the clinical trials conducted in PHC and 44.6 for the clinical trial conducted in the hospital, which were also comparable.

For the 2010–2015 period, the general indices of satisfaction with care were 91.8±9.5 for PHC centres versus 96.0±3.2 for SHC, showing statistically significant differences (*P* = 0.02); however, the values are above 80 points, which are classified as adequate.

In the 2016–2020 period, these values were higher than the previous period and without statistically significant differences (*P* = 0.07). For PHC centres, values of 94.4±8.0 were determined versus 97.9±1.5 for SHC.

The two outcome indicators also met the comparative standard and were assessed as adequate (Table 2).

## Discussion

### Summary

This is the first study in Cuba that has evaluated indicators of structure, process, and outcome in the implementation of a programme for conducting clinical oncology trials in PHC centres. The results provide novel data on the feasibility of its implementation.

Structure indicators showed that the programme had the adequate infrastructure, as well as the material and human resources required to meet the international quality standards required for these investigations.^17–20^

Process indicators reached comparable values with those obtained in phase III randomised clinical trial developed in the hospital for the following domains: ethical approval, patient retention, protocol compliance, and safety. Regarding the patient recruitment indicators, the study showed that PHC facilitated the recruitment of a greater number of patients in a shorter period of time, and the inclusion rates were greater than those achieved in SHC. Similarly, greater coverage of treated patients was achieved by reaching high proportions of patients treated in relation to the incidence of the disease.

In the research, the survival rates achieved for patients who received the product in PHC and SHC were similar. This indicator, tested in the group of patients who met the same inclusion criteria, guaranteed that the only difference was the level of medical care at which the patient was cared for. In addition, 'satisfaction with health care' was also comparable for patients enrolled in clinical trials in PHC and SHC, obtaining high levels of satisfaction in both scenarios. Therefore, these findings suggest that patients followed in the hospital by the oncologist showed survival and levels of satisfaction similar to those followed by the GPs in PHC together with the oncologist.

The experience also suggested that the PHC setting was adequate for the implementation of pragmatic clinical trials,^21^ with protocols with less stringent inclusion criteria,^22^ which combine simple variables that can be determined in PHC centres, with others that can be evaluated in SHC. To achieve this goal, a satisfactory integration between these levels of care is needed.

### Strengths and limitations

The main strength is that this longitudinal study used data spanning 10 years and included 17.2% of the total patients enrolled in the clinical trials.

There are some limitations. The clinical trial used as an external control was developed in an earlier period and a different setting, so the uptake indicators may be biased owing to the different conditions in which it was carried out.

Also, satisfaction with services in the clinical trial carried out at PHC could not be compared with the external control, because this aspect was not evaluated for a trial conducted at the hospital level. For this aspect, only the cross-sectional comparison was performed.

### Comparison with existing literature

Few references were found regarding programmes for conducting oncology clinical trials in PHC. Ersek *et al* published considerations on critical aspects to consider for the sustainability of a research programme based on oncology practices in the community. This study described only the basic elements of the programme and did not declare indicators for its evaluation. The present work is in line with the basic elements described by these authors,^5^ but it also proposes and evaluates feasibility indicators for the programme implementation. These indicators are representative of the fundamental aspects contained in the main international and national guidelines that regulate the conduct of clinical trials.^18–20^

The process indicators are consistent with those defined by Whitham *et al* in a study that validated indicators for the monitoring of multicentre randomised clinical trials using the Delphi methodology.^23^ The present study also defines indicators for the evaluation of processes and outcomes, using the Donabedian structure–process–outcome framework, which is recommended for the evaluation of real-world health interventions.^14^

The results obtained in the present study for the process indicators agree with those reported by other authors. The time for ethical approval of the protocol and modifications met the standard established by international regulations.^19,20,24^ It was determined that the incorporation of PHC into the trials can facilitate the recruitment of a greater number of patients, similar to that reported by Orzano *et al*.^25^ On the other hand, the rate of patients with lung cancer enrolled in the trials, in relation to the incidence of the disease (14.3%), was higher than this indicator in the Netherlands (7.6%).^26^ In addition, the evidence of product safety in the PHC setting and real-world conditions were consistent with findings from the previous phase III trial conducted in hospitals.^27^

Similarly, the high levels of satisfaction with services obtained in both settings are similar to those obtained by Grundfeld *et al* in a randomised trial comparing the satisfaction of patients with breast cancer followed in specialised institutions versus primary care. The study showed that some patients prefer to be followed in a setting close to their home, work, and family.^28^

The present programme promotes protocols in line with the 'shared model' proposed by American and European oncology societies, which include an initial intensive follow-up of the patient in the hospital for first-line oncology treatment, and then the patient is transferred to PHC to receive immunotherapy; maintaining periodic quarterly visits to the hospital.^29^

### Implications for practice

The programme has enabled the participation of patients with cancer in clinical trials conducted in their municipality of residence, facilitating better access to treatment with good adherence to the protocol.

It has also prepared the healthcare system for the administration of new therapies as part of routine medical practice, building capacity, and implementing work standards and routines to be used in practice. In addition, it will allow the implementation of other complex health interventions.^30^

## Data

The databases used in this study belong to different clinical trial protocols. The datasets generated during the current study are available from the corresponding author on reasonable request.
